# Sociodemographic predictors of digital health adoption in patients with asthma and COPD

**DOI:** 10.3389/fdgth.2025.1579983

**Published:** 2025-07-28

**Authors:** Daniela Téllez, Elroy Boers, Leanne Kaye, Vy Vuong, Meredith A. Barrett

**Affiliations:** ^1^ ResMed Science Center, San Diego, CA, United States; ^2^ ResMed Science Center, Halifax, NS, Canada

**Keywords:** digital health, social determinants of health, remote care, asthma, COPD

## Abstract

**Introduction:**

Remote healthcare adoption has grown significantly in United States (US). However, better characterization of patient behaviors and comfort with the use of digital health is needed, especially among vulnerable populations. The objective of this study was to evaluate how sociodemographic characteristics may relate to the adoption of digital health among patients with asthma and chronic obstructive pulmonary disease (COPD).

**Methods:**

Adults residing in the US and using a digital platform for asthma or COPD management were surveyed to understand (1) level of comfort sharing health data with their healthcare providers and (2) the presence of remote care concerns, specifically clinical, technological, privacy and financial concerns. Multivariable logistic regression models assessed the relationship between baseline disease status, sex, age, race, education, insurance, and income, with health data sharing patterns and areas of concern with remote care.

**Results:**

About one-third of survey respondents reported sharing health data with their provider, and most respondents had at least one concern in receiving remote care, with clinical and privacy concerns being the most frequently reported. However, attitudes and behaviors toward virtual health delivery were mixed. Patients with lower income were 65% more likely to share any health data (OR 1.65, 95% CI 1.13–2.43, *p* = 0.0104), but were also more likely to report at least one concern on virtual healthcare delivery. White patients (vs. non-white) were 2.5 times more likely to report clinical concerns when obtaining remote care (OR 2.5 95% CI 1.57–3.96, *p* = 0.0001).

**Discussion:**

Patterns of data sharing and concerns with remote care varied across sociodemographic predictors, sometimes in opposing ways. These learnings highlight the need for further research, including moderating and mediating factors like social support, health literacy, and rurality, to uncover the real-world use and impact of digital health services at a population level.

## Introduction

Asthma and chronic obstructive pulmonary disease (COPD) are chronic respiratory illnesses affecting over 600 million people worldwide (1–3), with increasing morbidity, mortality, and economic burden projections for the next decades (3, 4). Digital health programs focused on asthma and COPD disease management and exacerbation prevention have shown promising evidence in reducing healthcare utilization and hospitalizations (5, 6) improving medication adherence and disease control (7, 8), and empowering patients in the management of their chronic condition (9). These programs often include tools to facilitate patient-provider communication and disease management through medication tracking, symptom monitoring, and personalized insights from medication and/or therapy use that may help in early detection of exacerbations. However, these chronic conditions disproportionately affect older adults, individuals with low income, and racial/ethnic minorities, groups which are often underrepresented in digital health research and programs (10).

While prior studies have demonstrated the benefits of remote patient monitoring in improving adherence and reducing exacerbations in chronic respiratory illness (11), limited data exist on the perceived usability, concerns, and trust in these tools among socioeconomic and demographically diverse populations. Further, there is evidence that socioeconomic status (SES) and educational inequalities may negatively impact the benefit and user experience of digital health solutions (12, 13). This is commonly referred to as the digital divide, which has been also shown to be associated with health literacy, age, and rurality (14). These findings suggest that sociodemographic characteristics may influence the way which patients can obtain the maximum value out of digital health programs beyond access. It is thus important to distinguish behavioral (i.e., usage of a mobile app), vs. perceptual (i.e., comfort level, favorable attitudes) benefits and concerns of digital health programs when conducting research aimed at closing gaps in health equity. Understanding how sociodemographic characteristics relate to patients' relationships to digital tools is essential for tailoring inclusive, effective interventions.

As such, in the context of COPD and asthma care management, there is an opportunity to better understand patients' perception, attitudes, and behaviors in using their digital health tools. Furthermore, understanding areas of concern, and the willingness to share health data may inform the optimization of tools, workflows, and programs to maximize the improvement of health outcomes among more vulnerable and underserved populations. This analysis aimed to evaluate the sociodemographic factors and clinical characteristics associated with the likelihood of sharing digital health data with healthcare providers and the likelihood of having concerns about virtual healthcare delivery.

## Materials and methods

This retrospective observational study analyzed data from an electronic survey sent to a cohort of adult users (18+ years) of a digital health platform for asthma and COPD management (Propeller Health) from April-May 2021. A detailed description of the Propeller Health platform has been previously described, but in short, it is comprised of a medication sensor to track inhaler usage and a patient-facing mobile application (app) to share educational materials and insights on mediation usage trends (15, 16).

The survey was reviewed and approved by the Western Institutional Review Board (WIRB) (reference number 1300003) and all respondents provided electronic consent prior to survey initiation. The 15-item survey included multiple choice questions about behaviors, attitudes, and concerns in managing their asthma or COPD care remotely, as well as self-reported demographic and comorbidity data. All patients who completed the survey were included in the analysis.

Descriptive analyses were used to characterize the study population. Multivariable regression models were used to examine associations of self-reported sociodemographic and clinical characteristics (age, gender, income, insurance type, education, and asthma or COPD diagnosis, condition burden) that may be associated with health data sharing and remote care concerns (15, 16).

Modeling covariates were dichotomized as follows: lower education was defined as high school graduate or lower, and lower income was defined as an annual income of <49,999 USD. Disease burden was defined by the baseline Asthma Control Test (ACT) score for patients with asthma, or the COPD Assessment Test (CAT) score for patients with COPD. An ACT score ≤19 and a CAT score >20 corresponded to greater disease burden, respectively.

Health data sharing and the presence of any concern were the dichotomous outcomes used for the models. Health data sharing was defined as user-reported sharing of digital platform data (e.g., medication use, reports, symptoms, triggers) or other health data (e.g., vitals, oximetry, physical activity, prescriptions) with a healthcare provider. Areas of concern about using remote tools to obtain care or communicate with their health provider were categorized into financial, technological/logistical, clinical, privacy, or “other” concern type, and dichotomized for each category (e.g., technological concern or not). Multivariable logistic regressions models were developed to examine the outcomes of interest. A stepwise regression approach was then used for model selection, leveraging an iterative process to select the best model fit based on AIC and BIC. Odds ratios (OR) values are reported with 95% confidence intervals (CI) and *P*-values. A significance threshold of 0.05 was applied for all analyses. Statistical analyses were performed using R software (version 4.3; R Foundation for Statistical Computing, Vienna, Austria).

## Results

The analysis included 556 patients, of whom 76.3% reported an asthma diagnosis and 23.7% reported a COPD diagnosis. The mean (SD) age of the analyzed population was 52.5 (15.1) years old, with almost 40% over the age of 60 years. The study population was 66.4% female, 78.4% white, 54.1% privately insured. Additionally, 62.9% reported an income above 50,000 USD per year, and 87.1% were college graduates or above. Almost 60% of patients were considered to have a high burden of disease (Table 1).

**Table 1:** Baseline demographic characteristics (*n* = 556).

Variable	*N* (%)
Asthma	424 (76.3)
COPD	132 (23.7)
Age (mean ± SD)	52.5 ± 15.1
≥60 years old	216 (38.8)
Sex	
Male	187 (33.6)
Race	
White	436 (78.4)
Black or African American	51 (9.2)
Hispanic or Latino	36 (6.5)
Asian or Asian American	13 (2.3)
American Indian or Alaska Native	21 (3.8)
Other	5 (0.9)
Prefer not to answer	15 (2.7)
Education	
Some high school or less	8 (1.4)
High school graduate/ GED	64 (11.5)
Some college or technical school	169 (30.4)
College graduate	158 (28.4)
Graduate or professional degree	120 (21.58)
Prefer not to answer	10 (1.8)
Insurance	
Medicaid/CHIP	55 (9.9)
Medicare	130 (23.4)
Private health insurance	318 (57.2)
Uninsured	10 (1.8)
Other public insurance	10 (1.8)
Income	
<14,999 USD	59 (10.6)
15,000–24,999 USD	62 (11.2)
25,000–49,999 USD	85 (15.3)
50,000–74,999 USD	72 (12.9)
75,000–99,999 USD	44 (7.9)
>100,000 USD	107 (19.2)
Prefer not to answer	100 (18.0)
Disease burden (mean ± SD)	
ACT score (*n* = 396)	15.9 (4.8)
CAT score (*n* = 125)	18.7 (7.7)
High disease burden	
ACT score ≤19	282 (66.5)
CAT score > 20	50 (40)

Data are presented as mean ± standard deviation, or no. (%). ACT, asthma control test; CAT, COPD, assessment score. All variables were dichotomized to facilitate analysis.

### Health data sharing

**Figure 1:**
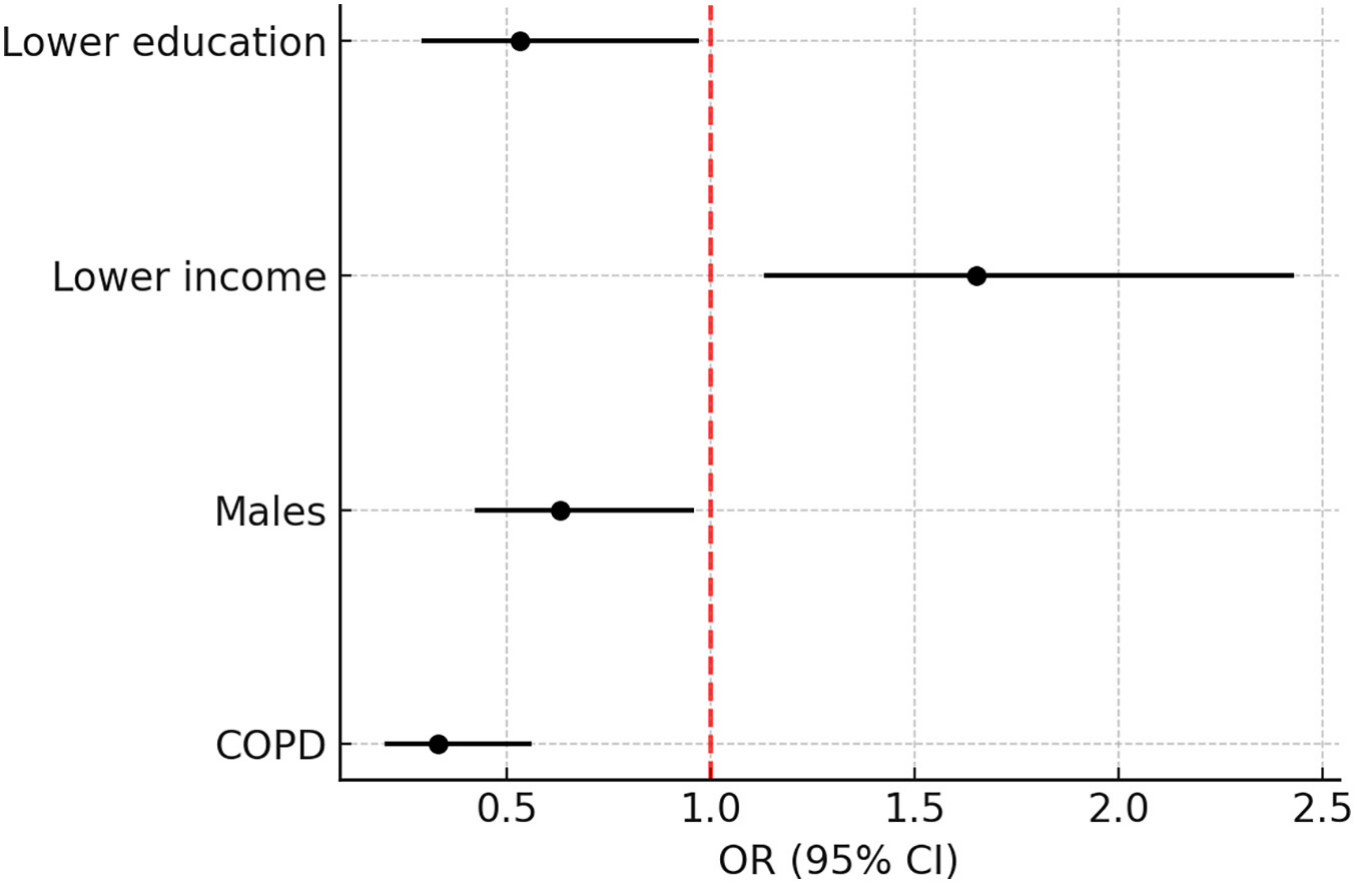
Adjusted odds ratios for health data sharing.

Among the features tracked on the digital health app, rescue medication use and monthly insights reports were the most frequently shared with healthcare providers. In addition, patients also reported sharing health data not captured by the platform, such as oximetry levels, prescriptions, and heart rate data (Table 2). Lower income patients were 65% higher odds to share any health data with their providers (OR 1.65, 95% CI 1.13–2.43, *p* = 0.010), while male patients and patients with a lower education level had decreased odds of sharing any health data (OR 0.63 95% CI 0.42–0.96, *p* = 0.032; OR 0.53, 95% CI 0.29–0.97, *p* = 0.038, respectively) (Figure 1). Patients with COPD had lower odds of health data sharing when compared to patients with asthma (OR 0.33, 95% CI 0.20–0.56, *p* = 0.0002) (Table 3).

**Table 2:** Health data sharing features included in the study questionnaire.

Sharing Features	*N* (%)
Propeller Health Features	
Controller medication	49 (8.81)
Rescue medication	72 (12.94)
Monthly report	61 (10.97)
Symptoms	25 (4.49)
Triggers	24 (4.31)
Other Health Data	
Breath rate	25 (4.49)
Heart rate	66 (11.87)
Oximetry	75 (13.49)
Physical activity	47 (8.45)
Prescriptions	66 (11.87)

**Table 3:** Odds for health data sharing among patients with asthma and COPD (*n* = 556).

Predictor	*β* Estimate	OR (95% CI)	*p*-value
COPD (Ref: Asthma)	−1.10	0.33 (0.20, 0.56)	0.00002
Males (Ref: Females)	−0.46	0.63 (0.42, 0.96)	0.0322
Lower income (Ref: Higher income)	0.50	1.65 (1.13, 2.43)	0.0104
Lower education (Ref: Higher education)	−0.63	0.53 (0.29, 0.97)	0.0381

### Concerns about remote care

About one-third of patients expressed having no concerns about remote asthma or COPD care (Table 4). Of those reporting concerns, the most common concern revolved around clinical aspects of remote care, including the quality of care received or ensuring that healthcare providers understood their needs (42.3%). Technological and logistic barriers of remote care, such as troubleshooting access or scheduling, were expressed as a concern among 35.1% of patients. A smaller proportion of the survey respondents noted financial concerns (8.3%) and privacy concerns (7.2%).

**Table 4:** Areas of concern for receiving remote care.

Concern Type	Survey Response	*N* (responses)	%
Financial	Financial/copay	46	8.3
Technological/Logistical	Provider availability or scheduling	108	35.1
	Feels complicated or stressful	40	
	Technology or access issues	86	
Clinical	Quality of care	158	42.3
	If my doctor understands my needs	134	
	Physical visit needed for asthma or COPD tests/assessment	17	
Privacy	Privacy	40	7.2
Other	Not offered by provider	3	0.9
	Not interested	2	
None	No concerns	151	27.2

### Clinical concerns

**Figure 2:**
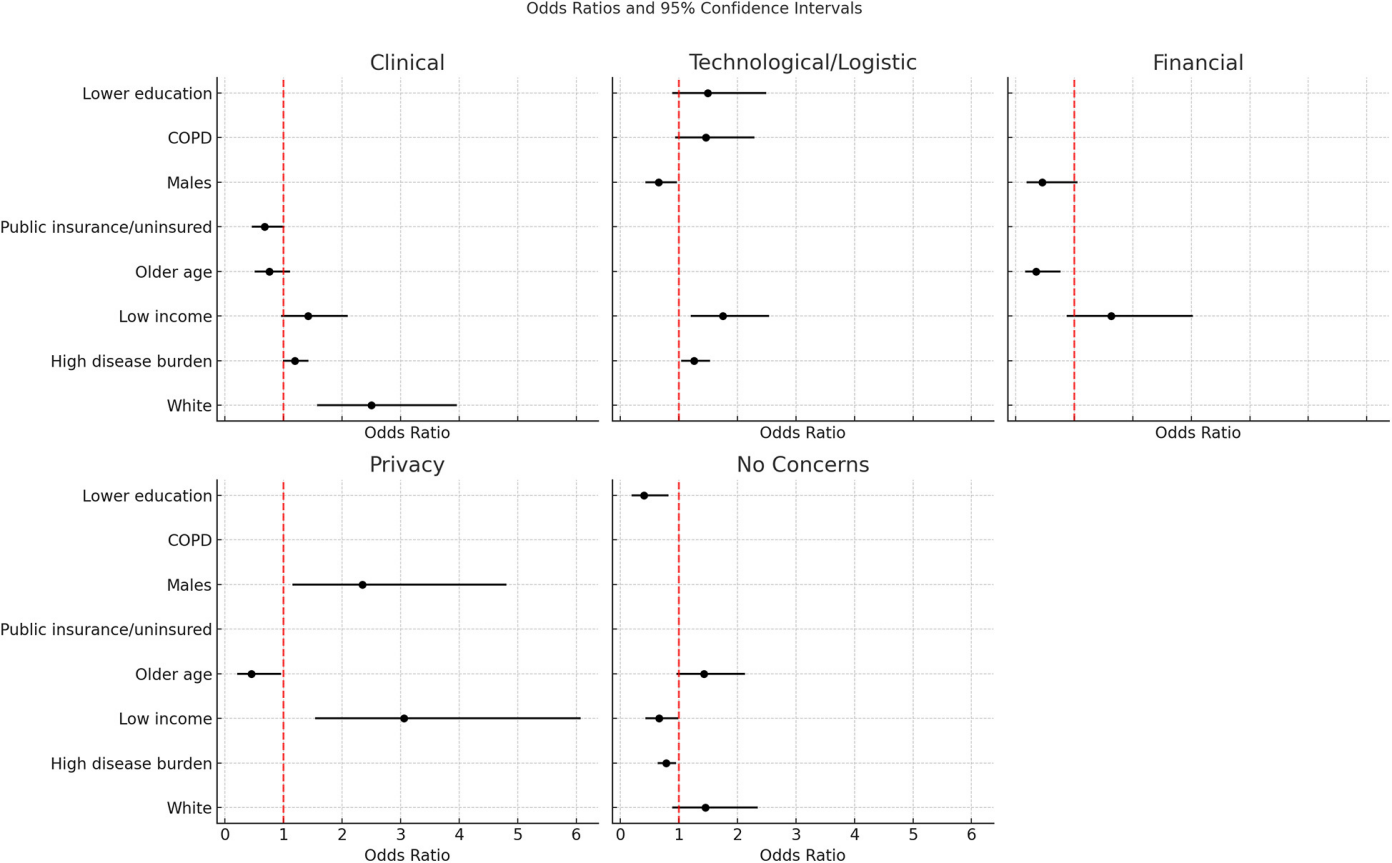
Adjusted odds ratios for identified concerns in remote care.

White patients (vs. non-white) presented 2.5 the odds to report clinical concerns when obtaining remote care (OR 2.5, 95% CI 1.57–3.96, *p* = 0.0001). Race was the only statistically significant predictor when modeling clinical concern about receiving remote care, but higher disease burden and low income trended towards increased odds of concern (OR 1.19, 95% CI 0.99–1.43, *p* = 0.068; OR 1.42, 95% CI 0.96–2.10, *p* = 0.073, respectively). Older age and having public insurance were associated with a trend towards decreased odds for having clinical concerns (OR 0.76, 95% CI 0.51–1.11, *p* = 0.153; OR 0.68, 95% CI 0.46–1.02, *p* = 0.065; respectively) (Table 5; Figure 2).

**Table 5:** Odds for areas of concern regarding remote care delivery (*n* = 556).

Clinical Concerns			
Predictor	β Estimate	OR (95% CI)	*p*-value
White (Ref: Any other race)	0.92	2.50 (1.57, 3.96)	0.0001
High disease burden (Ref: Low or moderate disease burden)	0.17	1.19 (0.99, 1.43)	0.068
Low income (Ref: Higher income)	0.36	1.42 (0.96, 2.10)	0.073
Older age (Ref: <60 years old)	−0.27	0.76 (0.51, 1.11)	0.153
Public insurance or uninsured (Ref: Private insurance)	−0.37	0.68 (0.46, 1.02)	0.065
Technological/Logistic Concerns			
Predictor	β Estimate	OR (95% CI)	*p*-value
Low income (Ref: Higher income)	0.56	1.75 (1.20, 2.54)	0.003
High disease burden (Ref: Low or moderate disease burden)	0.23	1.26 (1.04, 1.53)	0.020
Males (Ref: Females)	−0.44	0.65 (0.43, 0.97)	0.036
COPD (Ref: Asthma)	0.38	1.46 (0.94, 2.29)	0.095
Lower education (Ref: Higher education)	0.44	1.49 (0.89, 2.49)	0.128
Financial Concerns			
Predictor	β Estimate	OR (95% CI)	*p*-value
Older age (Ref: <60 years old)	−1.06	0.35 (0.16, 0.77)	0.009
Males (Ref: Females)	−0.80	0.45 (0.19, 1.06)	0.067
Low income (Ref: Higher income)	0.49	1.63 (0.87, 3.03)	0.125
Privacy Concerns			
Predictor	β Estimate	OR (95% CI)	*p* - value
Low income (Ref: Higher income)	1.12	3.06 (1.54, 6.08)	0.001
Males (Ref: Females)	0.86	2.35 (1.15, 4.81)	0.018
Older age (Ref: <60 years old)	−0.80	0.45 (0.21, 0.96)	0.037
No Concerns			
Predictor	β Estimate	OR (95% CI)	*p*-value
Lower education (Ref: Higher education)	−0.91	0.40 (0.19, 0.82)	0.012
High disease burden (Ref: Low or moderate disease burden)	−0.25	0.78 (0.64, 0.95)	0.014
Low income (Ref: Higher income)	−0.42	0.66 (0.43, 0.99)	0.049
Older age (Ref: <60 years old)	0.36	1.43 (0.96, 2.13)	0.075
White (Ref: Any other race)	0.37	1.45 (0.89, 2.35)	0.136

### Technological or logistical concerns

Lower income and higher burden of disease were associated with increased technological or logistic concerns (OR 1.75, 95% CI 1.20–2.54, *p* = 0.003; OR 1.26, 95% CI 1.04–1.53, *p* = 0.02, respectively). A COPD diagnosis (vs. asthma) and lower education also had higher odds of reporting technology or logistic concerns, although the confidence intervals were wide and included the null, indicating imprecision and uncertainty in these findings (OR 1.46, 95% CI 0.94–2.29, *p* = 0.095; OR 1.49, 95% CI 0.89–2.49, *p* = 0.128, respectively). Conversely, males reported decreased odds to have these concerns (OR 0.65, 95% CI 0.43–0.97, *p* = 0.036) (Table 5; Figure 2).

### Financial concerns

Older age was associated with 65% lower likelihood of financial concerns (OR 0.35, 95% CI 0.16–0.77, *p* = 0.009). Although not statistically significant, males also presented decreased odds for financial concerns (OR 0.45, 95% CI 0.19–1.06, *p* = 0.67). Lower income patients were 63% more likely to express financial concerns (OR 1.63 95% CI 0.87–3.03, *p* = 0.125) but this did not reach statistical significance in this sample (Table 5; Figure 2).

### Privacy concerns

Older patients were 55% less likely to express privacy concerns (OR 0.45, 95% CI 0.21–0.96, *p* = 0.037). Lower income was associated with more than 3 times the odds of privacy concerns (OR 3.06, 95% CI 1.54–6.08, *p* = 0.001). Males were more than twice as likely to have privacy concerns than females (OR 2.35, 95% CI 1.15–4.81, *p* = 0.018) (Table 5; Figure 2).

### Any concerns vs. no concerns

Older patients and white patients presented higher odds of reporting no concerns (OR 1.43, 95% CI 0.96–2.13, *p* = 0.075; OR 1.42, 95% CI 0.89–2.35, *p* = 0.136, respectively), although these did not reach statistical significance. Regression results showed that greater disease burden, lower income, and lower education were less likely to have no concerns with remote care. In other words, patients with lower income or education or higher disease burden were more likely to have at least one area of concern with remote healthcare delivery. Patients with greater disease burden were 22% less likely to report no concerns (OR 0.78, 95% CI 0.64–0.95, *p* = 0.014), patients with lower education were 60% less likely to report no concerns (OR 0.40, 95% CI 0.19–0.82, *p* = 0.012), and patients with lower income were 34% less likely to report no concerns (OR 0.66, 95% CI 0.43–0.99, *p* = 0.049) (Table 5; Figure 2).

## Discussion

This study reports a nuanced investigation of sociodemographic predictors and digital health adoption among patients using a digital health platform for asthma and/or COPD management. Approximately 30% of survey respondents reported no concerns with remote care; however, of concerns reported, clinical, technological and logistical were the most frequent, while financial and privacy concerns were the least frequently reported (<9% for both). Concern with remote care and likelihood of data sharing varied significantly by sociodemographic predictors including income, education, race, age and gender, as well as disease burden. Some characteristics including income, education, and age had opposing trends in areas of concern and health data sharing behaviors with digital health. For example, lower income individuals were significantly associated with increased odds of multiple areas of concern, but they were also the most likely to share digital health data with providers. These associations suggest complex behaviors and patterns in how populations adopt and benefit from digital health services.

The likelihood of any type of concern about remote care was higher among patients reporting lower income and poorer disease status, which may be explained in part by the link between socioeconomic status and higher risk of disease exacerbations (17). These concerns may stem from clinical factors, as patients at higher risk of exacerbations and with a greater impact of their condition on daily life may be more concerned about accessing timely care. Additionally, lower-income patients have been shown to express insecurities in effectively communicating their needs to providers (18) and understanding digital health solutions (19), which may add stress and uncertainty to healthcare interactions and disease management. This observed outcome may further be connected to lower health literacy and the technological and logistical concerns also observed among lower income patients. On the other hand, healthcare providers have similarly reported difficulty identifying and communicating with low health literate patients (20). Thus, it is important to provide training and establish communication lines in digital health platforms to ensure comfort across patients of all levels of health literacy.

The clinical concerns described in this study may reflect gaps in trust and confidence in remote and digital health tools. Results suggest that there is continued reliance on traditional in-person visits for asthma and COPD management. For example, physical tests like spirometry or x-ray imaging were written as a frequent free-text response by survey respondents. White patients in this sample had the strongest association for greater odds of clinical concerns compared to any other race. While this may have been largely driven by the predominantly White sample, this association may be reflective of the patient experience and expectations when receiving care. Previous studies have identified racial disparities in the access and usage of telehealth services, with Black and Hispanic patients being less likely to use these tools and have fewer total health visits (both in-person and remote) as compared to White patients (21). These data help explain the larger proportion of White patients in this sample and may point to areas of improvement for remote care delivery by patients who are established and consistent users.

In previous research, barriers to digital health adoption have been identified for older adults (22); however, results from this study showed older adults were at lower odds of reporting any concern in using remote care. Several factors may contribute to this observation beyond convenience and accessibility. For example, the COVID-19 pandemic increased awareness of infection risk through in-person visits, especially among older age patients (23). Remote care became the safer alternative to minimize exposures and heightened the importance of technology to connect with loved ones and healthcare providers. An increase in technology adoption was seen among older adults, with reports of 42% of older adults viewing technology more positively compared to pre-pandemic times (24). This collective introduction (or re-introduction) to technology and digital tools may have contributed to reducing odds for concerns in using digital health management platforms and obtaining virtual healthcare.

Available data indicates gender differences in the adoption of telehealth services, showing that females (including patients and physicians) are more likely to use digital health tools (25). While the majority of this study's sample was female, males stood out with decreased odds of technology and financial concerns with remote care, but more concern about privacy issues. No current research has identified gender disparities in privacy concerns, but a systematic review conducted in 2022 highlighted general privacy and security challenges sparked by the rapid uptake of digital health services from the COVID-19 public health emergency (26). Further research is needed to discern gender specific perceptions on digital health and to develop best practices for tailored telehealth delivery.

While the results from this study point to the need for additional investigation into the role of sociodemographic characteristics in comfort with data sharing and concerns about remote care, these results should be interpreted in light of the study's limitations. First, our sample population only included patients who were active users of an asthma and COPD digital health management platform. Data on initial access to the platform, frequency of platform use, health literacy, and other factors that may influence the relationship to digital health platforms were not available for analysis. Second, the confidence intervals for some results were wide, indicating that the true effect could range from a modest decrease to a substantial increase in odds. This imprecision underscores the need for further research with a larger sample size to obtain more stable estimates and validate these associations. Third (27), while the study sample was largely White, African Americans comprised about 10% of the sample, a little less than the national average of 13.7% within the United States (28). However, the sample likely does not sufficiently reflect the racial and ethnic inequities within asthma and COPD, which often show higher prevalence rates and/or greater disease burden among non-white populations (29, 30). That said, the study did include a more diverse sample across age, education, household income and insurance coverage. Fourth, the survey was cross-sectional in nature, and was collected during the COVID-19 pandemic, so it was limited in capturing patient beliefs and experiences over time, such as before vs. after the pandemic. Lastly, the survey design had a notable limitation in that it did not include a “None” or “Not Applicable” response option for the question regarding concerns about remote care. As a result, respondents who indicated having no concerns in the open text “Other” field were categorized as having no concerns. While this approach allowed for the capture of some respondents' true sentiments, the absence of an explicit “None” option may have introduced ambiguity or response bias, as participants without concerns may have skipped the question or failed to clarify their position. More robust statistical methods like structural equation modeling may also offer clearer and more precise results into sociodemographic relationships with health data sharing behaviors and concerns.

## Conclusion

The relationship between digital health and patient receptiveness is nuanced and evolving. Data from this analysis provides meaningful insights into how patients with chronic respiratory conditions perceive and interact with digital health solutions, and how they can vary distinctly by sociodemographic characteristics. Results were consistent with available literature in reinforcing the association between sociodemographic vulnerabilities like lower income and higher disease burden to increased odds of concerns about virtual health delivery. However, this study challenges previous findings about older age patients and their comfort with digital health and uncovers complex associations that point to the intersectionality of sociodemographic predictors and attitudes about digital health. Results point to the vital need for further research, including moderating and mediating factors like social support, health literacy, and rurality, to uncover the real-world use and impact of digital health services at a population level and further reinforce the need to include patients who have been historically underrepresented in digital health research. Data from these future studies can inform providers and product designers methods to narrow the digital divide by understanding population needs and then tailoring interventions to optimize the patient experience and health-related quality of life outcomes for all patients with chronic conditions (5).

## Data Availability

The raw data supporting the conclusions of this article will be made available by the authors, without undue reservation.
